# 4,4′-Bipyridine–2-hydroxy­propane-1,2,3-tricarboxylic acid (3/2)

**DOI:** 10.1107/S1600536809004607

**Published:** 2009-02-18

**Authors:** Janet Soleimannejad, Hossein Aghabozorg, Shokoh Najafi, Mina Nasibipour, Jafar Attar Gharamaleki

**Affiliations:** a Department of Chemistry, Faculty of Science, Ilam University, Ilam, Iran; b Faculty of Chemistry, Tarbiat Moallem University, 49 Mofateh Avenue, 15614 Tehran, Iran

## Abstract

The combination of 2-hydroxy­propane-1,2,3-tricarboxylic acid (H_3_hypta, also called citric acid) and 4,4′-bipyridine (4,4′-bipy) in a 1:1.5 molar ratio leads to the formation of the title mol­ecular cocrystal, 1.5C_10_H_8_N_2_·C_6_H_8_O_7_. The asymmetric unit contains one and a half 4,4′-bipy units, with one lying across a centre of inversion, and one H_3_hypta mol­ecule. The significant differences in the C—O bond distances support the existence of the un-ionized acid mol­ecule and confirm the formation of a cocrystal. There are π–π and C—H⋯π stacking inter­actions between the aromatic rings of 4,4′-bipy [with inter­planar distances of 3.7739 (8) and 3.7970 (8) Å] and between CH groups of H_3_hypta [with an H⋯π distance of 2.63 Å]. In the crystal structure, intermolecular O—H⋯N hydrogen bonds occur and an O—H⋯O hydrogen bond occurs within the citric acid moiety.

## Related literature

For related literature on cocrystals and hydrogen bonding, see: Aakeroy & Seddon (1993 ▶); Aghabozorg *et al.* (2006 ▶); Aghabozorg, Heidari *et al.* (2008 ▶); Aghabozorg, Manteghi & Sheshmani (2008 ▶); Baures (1999 ▶); Biradha *et al.* (1993 ▶); Desiraju (1989 ▶); Desiraju & Steiner (1999 ▶); Houk *et al.* (1999 ▶).
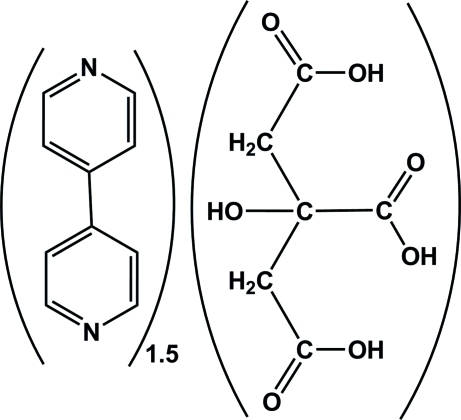


## Experimental

### 

#### Crystal data

1.5C_10_H_8_N_2_·C_6_H_8_O_7_
*M* *_r_* = 426.40
Monoclinic, 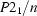

*a* = 7.0371 (2) Å
*b* = 33.5054 (10) Å
*c* = 8.4715 (2) Å
β = 90.302 (2)°
*V* = 1997.39 (9) Å^3^
*Z* = 4
Mo *K*α radiation
μ = 0.11 mm^−1^
*T* = 150 K
0.31 × 0.26 × 0.22 mm

#### Data collection

Bruker SMART CCD area-detector diffractometer
Absorption correction: multi-scan (*SADABS*; Bruker, 2005 ▶) *T*_min_ = 0.896, *T*_max_ = 0.977
63749 measured reflections
6045 independent reflections
4986 reflections with *I* > 2σ(*I*)
*R*_int_ = 0.033

#### Refinement

*R*[*F* ^2^ > 2σ(*F* ^2^)] = 0.045
*wR*(*F* ^2^) = 0.116
*S* = 1.05
6045 reflections
284 parameters
H-atom parameters constrained
Δρ_max_ = 0.40 e Å^−3^
Δρ_min_ = −0.24 e Å^−3^

### 

Data collection: *SMART* (Bruker, 2007 ▶); cell refinement: *SAINT* (Bruker, 2007 ▶); data reduction: *SAINT*; program(s) used to solve structure: *SHELXS97* (Sheldrick, 2008 ▶); program(s) used to refine structure: *SHELXL97* (Sheldrick, 2008 ▶); molecular graphics: *SHELXTL* (Sheldrick, 2008 ▶); software used to prepare material for publication: *SHELXTL*.

## Supplementary Material

Crystal structure: contains datablocks I, global. DOI: 10.1107/S1600536809004607/su2092sup1.cif

Structure factors: contains datablocks I. DOI: 10.1107/S1600536809004607/su2092Isup2.hkl

Additional supplementary materials:  crystallographic information; 3D view; checkCIF report

## Figures and Tables

**Table 1:** Hydrogen-bond geometry (Å, °)

*D*—H⋯*A*	*D*—H	H⋯*A*	*D*⋯*A*	*D*—H⋯*A*
O1—H1*A*⋯N3^i^	0.84	1.80	2.6275 (14)	168
O3—H3*A*⋯N1^ii^	0.84	1.75	2.5880 (14)	173
O6—H6*A*⋯N2^iii^	0.84	1.82	2.6443 (14)	168
O7—H7*A*⋯O4	0.84	2.22	2.6538 (13)	112
C2—H2*A*⋯*Cg*^iv^	0.99	2.63	3.5579 (13)	160

Symmetry codes: (i) 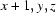; (ii) 
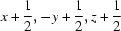
; (iii) 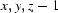; (iv) 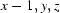. *Cg* is the centroid of the N3,C17–C21 ring.

**Fig. 1.**
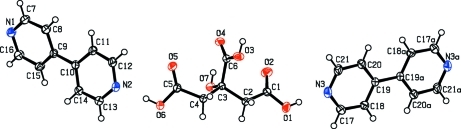
The molecular structure of the title compound, showing the displacement ellipsoids drawn at the 50% probability level. [The suffix a denotes atoms generated by the symmetry operator (-x, -y, -z + 1).

**Fig. 2.**
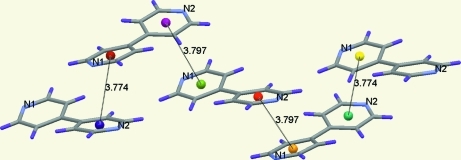
A view of the π–π stacking interaction between two Cg1 and Cg2 aromatic rings of the 4,4'-bipyridine units, with distances of 3.7739 (8) Å; 1/2 + x, 1/2 - y, -1/2 + z and 3.7970 (8) Å; -1/2 + x, 1/2 - y, -1/2 + z [Cg1 and Cg2 are centroids for the aromatic rings N1/C7–C9/C15/C16 and N2/C10–C14, respectively].

**Fig. 3.**
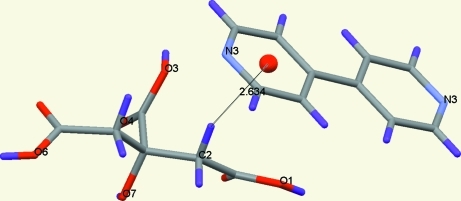
A view of the C—H···π stacking interactions between CH groups of 2-hydroxypropane-1,2,3-tricarboxylic acid with the aromatic rings of 4,4'-bipyridine: H···π distance of 2.634 Å for C2—O2A···Cg3 (-1 + x, y, z) [Cg3 is the centroid of ring N3/C17–C21].

**Fig. 4.**
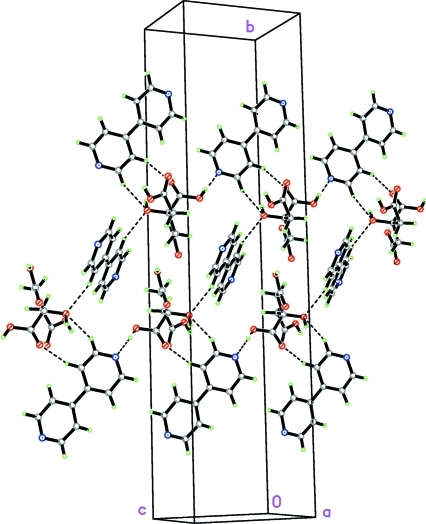
Two 4,4'-bipy and H3hypta fragments are linked together by O—H···N and C—H···O hydrogen bonds.

**Fig. 5.**
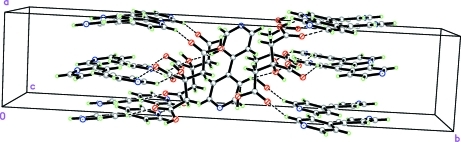
Crystal packing of the title compound viewed down the c axis (hydrogen bonds are shown as dashed lines).

## supplementary crystallographic information

### Comment

The creation of new functional materials through the control of intermolecular bonding is a key aim of crystal engineering (Desiraju, 1989). The synthesis of crystalline supramolecular structures mediated by hydrogen bonds is of considerable importance. Among all the non-bonded interactions, hydrogen bonding has proved to be the most useful and reliable, because of its strength and directional properties (Aakeroy & Seddon, 1993; Aghabozorg, Heidari *et al.*, 2008).

In the case of cocrystals, these are generally formed by dissolution and recrystallization from a suitable solvent, although sublimation and growth from the melt are also used. Co-crystallization is a deliberate attempt at bringing together different molecular species in one crystalline lattice without making or breaking covalent bonds (Aghabozorg *et al.*, 2006). Cocrystals are used to reveal specific recognition motifs, such as those proposed for rational drug design (Baures, 1999; Houk *et al.*, 1999) and crystal engineering applications.

The asymmetric unit of the title cocrystal is shown in Fig. 1, and geometrical parameters are availabe in the archived CIF. The asymmetric unit contains one and a half 4,4'-bipy units and one H_3_hypta molecule. One 4,4'-bipy unit is located on a center of inversion. The C—O distances support the existence of the unionized acid molecules, indicating cocrystal formation; the C1—O1 [1.3203 (15) Å], C5—O6 [1.3197 (15) Å] and C6—O3 [1.3045 (15) Å] bond lengths are significantly longer than the C1—O2 [1.2070 (16) Å], C5—O5 [1.2084 (15) Å] and C6—O4 [1.2131 (15) Å] bond lengths.

The dihedral angle involving the aromatic rings, N1/C7–C9/C15/C16 (*Cg*1) and N2/C10–C14 (*Cg*2), of a 4,4'-bipy is 18.67°, which shows these units are not in the same plane, and also indicates the flexibility of the central C—C bond.

As shown in Fig. 2, there are π–π stacking interactions between two aromatic rings, *Cg*1 and *Cg*2, of the 4,4'-bipy units, with distances of 3.7739 (8) Å [1/2 + *x*, 1/2 - *y*, -1/2 + *z*] and 3.7970 (8) Å [-1/2 + *x*, 1/2 - *y*, -1/2 + *z*]. It can be seen in Fig. 3, that there are also C—H···π stacking interactions between CH groups of 2-hydroxypropane-1,2,3-tricarboxylic acid and the aromatic rings of 4,4'-bipyridine, with an H···π distance of 2.63 Å for C2—O2A···*Cg*3 [-1 + *x*, *y*, *z;* where *Cg*3 is the centroid of ring N3/C17–C21].

A remarkable feature in the crystal structure of the title compound is the presence of a large number of O—H···O, O—H···N and C—H···O hydrogen bonds (Table 1). There is an intramolecular O7—H7A···O4 hydrogen bond between the hydroxyl group and the carboxylate carbonyl group of the H_3_hypta unit, with distance D···A of 2.6538 (13) Å. Two 4,4'-bipy and H_3_hypta fragments are linked together by O—H···N and C—H···O hydrogen bonds and form chains (Fig. 4). C—H···O hydrogen bonding is widely accepted (Desiraju & Steiner, 1999; Biradha *et al.*, 1993), and weak hydrogen bonding can be exploited in supramolecular chemistry and crystal structure design (Aghabozorg, Manteghi & Sheshmani, 2008). The crystal packing of the title compound is illustrated in Fig. 5.

### Experimental

An aqueous solution (50 ml) of 4,4'-bipyridine (100 mg, 6 mmol) and 84 mg (4 mmol) of 2-hydroxypropane-1,2,3-tricarboxylicacid, [H_3_hypta, also called citric acid] were refluxed for two hours. Yellow crystals of the title compound were obtained from the solution after a few weeks at room temperature.

### Refinement

The H atoms were included in calculated positions and treated as riding atoms: O—H = 0.84 Å, C—H = 0.95–0.99 Å, with U_iso_(H) = 1.2U_eq_(parent O or C atom).

### Crystal data

**Table:**

1.5C_10_H_8_N_2_·C_6_H_8_O_7_	*F*(000) = 892
*M**_r_* = 426.40	*D*_x_ = 1.418 Mg m^−^^3^
Monoclinic, *P*2_1_/*n*	Mo *K*α radiation, λ = 0.71073 Å
Hall symbol: -P 2yn	Cell parameters from 20381 reflections
*a* = 7.0371 (2) Å	θ = 2.7–29.6°
*b* = 33.5054 (10) Å	µ = 0.11 mm^−^^1^
*c* = 8.4715 (2) Å	*T* = 150 K
β = 90.302 (2)°	Block, yellow
*V* = 1997.39 (9) Å^3^	0.31 × 0.26 × 0.22 mm
*Z* = 4	

### Data collection

**Table:**

Bruker SMART CCD area-detector diffractometer	6045 independent reflections
Radiation source: fine-focus sealed tube	4986 reflections with *I* > 2σ(*I*)
graphite	*R*_int_ = 0.033
φ and ω scans	θ_max_ = 30.5°, θ_min_ = 1.2°
Absorption correction: multi-scan (*SADABS*; Bruker, 2005)	*h* = −9→10
*T*_min_ = 0.896, *T*_max_ = 0.977	*k* = −47→47
63749 measured reflections	*l* = −12→12

### Refinement

**Table:**

Refinement on *F*^2^	Primary atom site location: structure-invariant direct methods
Least-squares matrix: full	Secondary atom site location: difference Fourier map
*R*[*F*^2^ > 2σ(*F*^2^)] = 0.045	Hydrogen site location: inferred from neighbouring sites
*wR*(*F*^2^) = 0.116	H-atom parameters constrained
*S* = 1.05	*w* = 1/[σ^2^(*F*_o_^2^) + (0.046*P*)^2^ + 1.026*P*] where *P* = (*F*_o_^2^ + 2*F*_c_^2^)/3
6045 reflections	(Δ/σ)_max_ < 0.001
284 parameters	Δρ_max_ = 0.40 e Å^−^^3^
0 restraints	Δρ_min_ = −0.24 e Å^−^^3^

### Special details

**Table:**

Geometry. All esds (except the esd in the dihedral angle between two l.s. planes) are estimated using the full covariance matrix. The cell esds are taken into account individually in the estimation of esds in distances, angles and torsion angles; correlations between esds in cell parameters are only used when they are defined by crystal symmetry. An approximate (isotropic) treatment of cell esds is used for estimating esds involving l.s. planes.
Refinement. Refinement of F^2^ against ALL reflections. The weighted R-factor wR and goodness of fit S are based on F^2^, conventional R-factors R are based on F, with F set to zero for negative F^2^. The threshold expression of F^2^ > 2sigma(F^2^) is used only for calculating R-factors(gt) etc. and is not relevant to the choice of reflections for refinement. R-factors based on F^2^ are statistically about twice as large as those based on F, and R- factors based on ALL data will be even larger.

### Fractional atomic coordinates and isotropic or equivalent isotropic displacement parameters (Å^2^)

**Table:**

	*x*	*y*	*z*	*U*_iso_*/*U*_eq_	
O1	1.22826 (14)	0.02331 (3)	0.66535 (13)	0.0327 (2)	
H1A	1.3359	0.0254	0.7078	0.049*	
O2	1.27436 (15)	0.08764 (3)	0.60893 (15)	0.0360 (3)	
O3	0.85874 (15)	0.12663 (3)	0.72640 (11)	0.0274 (2)	
H3A	0.8845	0.1464	0.7838	0.041*	
O4	1.00936 (14)	0.16212 (3)	0.54053 (11)	0.0258 (2)	
O5	0.59732 (15)	0.15627 (3)	0.37884 (11)	0.0274 (2)	
O6	0.52218 (17)	0.10777 (3)	0.20888 (12)	0.0339 (3)	
H6A	0.4824	0.1270	0.1541	0.051*	
O7	0.99435 (14)	0.10297 (3)	0.33252 (10)	0.02327 (19)	
H7A	1.0802	0.1200	0.3467	0.035*	
C1	1.17363 (18)	0.05848 (4)	0.61136 (14)	0.0207 (2)	
C2	0.97059 (17)	0.05761 (3)	0.55534 (15)	0.0195 (2)	
H2A	0.8884	0.0516	0.6468	0.023*	
H2B	0.9561	0.0354	0.4789	0.023*	
C3	0.89834 (17)	0.09596 (3)	0.47738 (13)	0.0173 (2)	
C4	0.68742 (18)	0.08896 (4)	0.43974 (15)	0.0208 (2)	
H4A	0.6741	0.0632	0.3832	0.025*	
H4B	0.6168	0.0867	0.5401	0.025*	
C5	0.59840 (18)	0.12151 (4)	0.34080 (14)	0.0206 (2)	
C6	0.92786 (18)	0.13235 (3)	0.58559 (14)	0.0194 (2)	
N1	0.42826 (18)	0.31593 (3)	0.42264 (13)	0.0263 (2)	
N2	0.42914 (18)	0.16451 (3)	1.00572 (14)	0.0278 (2)	
C7	0.4111 (2)	0.32444 (4)	0.57535 (16)	0.0285 (3)	
H7	0.3926	0.3515	0.6050	0.034*	
C8	0.4188 (2)	0.29590 (4)	0.69282 (15)	0.0262 (3)	
H8	0.4064	0.3034	0.8004	0.031*	
C9	0.44484 (18)	0.25603 (4)	0.65244 (14)	0.0206 (2)	
C10	0.44274 (18)	0.22416 (4)	0.77407 (14)	0.0203 (2)	
C11	0.4672 (2)	0.23319 (4)	0.93413 (15)	0.0262 (3)	
H11	0.4887	0.2599	0.9670	0.031*	
C12	0.4597 (2)	0.20272 (4)	1.04392 (16)	0.0291 (3)	
H12	0.4772	0.2093	1.1521	0.035*	
C13	0.4046 (2)	0.15593 (4)	0.85302 (16)	0.0275 (3)	
H13	0.3824	0.1289	0.8240	0.033*	
C14	0.4101 (2)	0.18445 (4)	0.73488 (15)	0.0243 (3)	
H14	0.3917	0.1769	0.6278	0.029*	
C15	0.4680 (2)	0.24726 (4)	0.49209 (15)	0.0262 (3)	
H15	0.4899	0.2206	0.4587	0.031*	
C16	0.4587 (2)	0.27789 (4)	0.38298 (15)	0.0282 (3)	
H16	0.4747	0.2716	0.2745	0.034*	
N3	0.56382 (16)	0.01882 (4)	0.80165 (13)	0.0261 (2)	
C17	0.6626 (2)	−0.01444 (4)	0.77585 (17)	0.0292 (3)	
H17	0.6137	−0.0332	0.7021	0.035*	
C18	0.83276 (19)	−0.02304 (4)	0.85097 (16)	0.0257 (3)	
H18	0.8979	−0.0472	0.8286	0.031*	
C19	0.90778 (16)	0.00392 (4)	0.95940 (13)	0.0183 (2)	
C20	0.8034 (2)	0.03831 (4)	0.98728 (16)	0.0274 (3)	
H20	0.8478	0.0575	1.0613	0.033*	
C21	0.6342 (2)	0.04450 (4)	0.90668 (17)	0.0295 (3)	
H21	0.5649	0.0682	0.9274	0.035*	

### Atomic displacement parameters (Å^2^)

**Table:**

	*U*^11^	*U*^22^	*U*^33^	*U*^12^	*U*^13^	*U*^23^
O1	0.0243 (5)	0.0270 (5)	0.0467 (6)	0.0001 (4)	−0.0148 (4)	0.0088 (4)
O2	0.0232 (5)	0.0249 (5)	0.0597 (7)	−0.0039 (4)	−0.0068 (5)	0.0009 (5)
O3	0.0403 (6)	0.0231 (5)	0.0190 (4)	−0.0043 (4)	0.0041 (4)	−0.0039 (3)
O4	0.0326 (5)	0.0186 (4)	0.0262 (5)	−0.0057 (4)	−0.0031 (4)	0.0012 (3)
O5	0.0372 (6)	0.0198 (4)	0.0250 (5)	0.0051 (4)	−0.0040 (4)	−0.0013 (3)
O6	0.0534 (7)	0.0212 (5)	0.0269 (5)	0.0017 (4)	−0.0190 (5)	0.0016 (4)
O7	0.0287 (5)	0.0223 (4)	0.0189 (4)	−0.0030 (3)	0.0044 (3)	−0.0009 (3)
C1	0.0199 (6)	0.0214 (6)	0.0207 (6)	0.0014 (4)	−0.0018 (4)	−0.0021 (4)
C2	0.0196 (6)	0.0158 (5)	0.0231 (6)	0.0000 (4)	−0.0042 (4)	0.0014 (4)
C3	0.0200 (6)	0.0153 (5)	0.0165 (5)	−0.0001 (4)	−0.0003 (4)	0.0000 (4)
C4	0.0208 (6)	0.0177 (5)	0.0238 (6)	−0.0007 (4)	−0.0053 (4)	0.0034 (4)
C5	0.0218 (6)	0.0200 (5)	0.0200 (6)	0.0016 (4)	−0.0011 (4)	0.0022 (4)
C6	0.0221 (6)	0.0172 (5)	0.0190 (5)	0.0012 (4)	−0.0030 (4)	−0.0008 (4)
N1	0.0366 (6)	0.0232 (5)	0.0193 (5)	0.0002 (4)	0.0017 (4)	0.0028 (4)
N2	0.0362 (6)	0.0247 (5)	0.0225 (5)	0.0027 (5)	−0.0070 (5)	0.0041 (4)
C7	0.0443 (8)	0.0197 (6)	0.0214 (6)	0.0031 (5)	0.0013 (5)	0.0003 (5)
C8	0.0405 (8)	0.0215 (6)	0.0164 (6)	0.0039 (5)	0.0013 (5)	−0.0005 (4)
C9	0.0239 (6)	0.0205 (5)	0.0175 (5)	0.0026 (4)	−0.0006 (4)	0.0008 (4)
C10	0.0216 (6)	0.0214 (6)	0.0178 (5)	0.0036 (4)	−0.0013 (4)	0.0010 (4)
C11	0.0379 (7)	0.0211 (6)	0.0196 (6)	0.0038 (5)	−0.0045 (5)	−0.0003 (4)
C12	0.0430 (8)	0.0255 (6)	0.0188 (6)	0.0047 (6)	−0.0061 (5)	0.0011 (5)
C13	0.0348 (7)	0.0216 (6)	0.0259 (6)	0.0005 (5)	−0.0072 (5)	0.0013 (5)
C14	0.0296 (7)	0.0234 (6)	0.0198 (6)	0.0014 (5)	−0.0037 (5)	−0.0009 (4)
C15	0.0389 (8)	0.0200 (6)	0.0198 (6)	0.0041 (5)	0.0028 (5)	−0.0019 (4)
C16	0.0419 (8)	0.0258 (6)	0.0168 (6)	0.0025 (5)	0.0037 (5)	−0.0005 (5)
N3	0.0197 (5)	0.0314 (6)	0.0272 (6)	0.0009 (4)	−0.0037 (4)	0.0068 (4)
C17	0.0244 (7)	0.0292 (7)	0.0338 (7)	−0.0025 (5)	−0.0087 (5)	−0.0018 (5)
C18	0.0229 (6)	0.0208 (6)	0.0333 (7)	0.0032 (5)	−0.0062 (5)	−0.0041 (5)
C19	0.0176 (5)	0.0203 (5)	0.0169 (5)	0.0015 (4)	−0.0009 (4)	0.0017 (4)
C20	0.0278 (7)	0.0268 (6)	0.0274 (6)	0.0086 (5)	−0.0065 (5)	−0.0057 (5)
C21	0.0277 (7)	0.0300 (7)	0.0306 (7)	0.0114 (5)	−0.0046 (5)	−0.0002 (5)

### Geometric parameters (Å, °)

**Table:**

O1—C1	1.3205 (15)	C8—C9	1.3915 (17)
O1—H1A	0.8400	C8—H8	0.9500
O2—C1	1.2073 (16)	C9—C15	1.4002 (17)
O3—C6	1.3048 (15)	C9—C10	1.4838 (17)
O3—H3A	0.8400	C10—C14	1.3902 (17)
O4—C6	1.2131 (15)	C10—C11	1.3990 (17)
O5—C5	1.2084 (15)	C11—C12	1.3820 (18)
O6—C5	1.3200 (15)	C11—H11	0.9500
O6—H6A	0.8400	C12—H12	0.9500
O7—C3	1.4235 (14)	C13—C14	1.3844 (18)
O7—H7A	0.8400	C13—H13	0.9500
C1—C2	1.5035 (17)	C14—H14	0.9500
C2—C3	1.5306 (16)	C15—C16	1.3825 (18)
C2—H2A	0.9900	C15—H15	0.9500
C2—H2B	0.9900	C16—H16	0.9500
C3—C4	1.5345 (17)	N3—C21	1.3313 (18)
C3—C6	1.5389 (16)	N3—C17	1.3323 (18)
C4—C5	1.5096 (16)	C17—C18	1.3832 (18)
C4—H4A	0.9900	C17—H17	0.9500
C4—H4B	0.9900	C18—C19	1.3906 (17)
N1—C7	1.3309 (17)	C18—H18	0.9500
N1—C16	1.3357 (17)	C19—C20	1.3875 (17)
N2—C13	1.3355 (17)	C19—C19^i^	1.489 (2)
N2—C12	1.3379 (18)	C20—C21	1.3851 (18)
C7—C8	1.3810 (18)	C20—H20	0.9500
C7—H7	0.9500	C21—H21	0.9500
			
C1—O1—H1A	109.5	C8—C9—C10	121.22 (11)
C6—O3—H3A	109.5	C15—C9—C10	121.63 (11)
C5—O6—H6A	109.5	C14—C10—C11	117.23 (11)
C3—O7—H7A	109.5	C14—C10—C9	121.67 (11)
O2—C1—O1	123.93 (12)	C11—C10—C9	121.06 (11)
O2—C1—C2	124.57 (11)	C12—C11—C10	119.19 (12)
O1—C1—C2	111.49 (10)	C12—C11—H11	120.4
C1—C2—C3	115.65 (10)	C10—C11—H11	120.4
C1—C2—H2A	108.4	N2—C12—C11	123.41 (12)
C3—C2—H2A	108.4	N2—C12—H12	118.3
C1—C2—H2B	108.4	C11—C12—H12	118.3
C3—C2—H2B	108.4	N2—C13—C14	123.22 (12)
H2A—C2—H2B	107.4	N2—C13—H13	118.4
O7—C3—C2	110.63 (10)	C14—C13—H13	118.4
O7—C3—C4	108.00 (9)	C13—C14—C10	119.55 (12)
C2—C3—C4	106.27 (9)	C13—C14—H14	120.2
O7—C3—C6	108.65 (9)	C10—C14—H14	120.2
C2—C3—C6	111.39 (9)	C16—C15—C9	119.18 (12)
C4—C3—C6	111.86 (10)	C16—C15—H15	120.4
C5—C4—C3	113.77 (10)	C9—C15—H15	120.4
C5—C4—H4A	108.8	N1—C16—C15	123.18 (12)
C3—C4—H4A	108.8	N1—C16—H16	118.4
C5—C4—H4B	108.8	C15—C16—H16	118.4
C3—C4—H4B	108.8	C21—N3—C17	117.24 (11)
H4A—C4—H4B	107.7	N3—C17—C18	123.37 (13)
O5—C5—O6	123.98 (11)	N3—C17—H17	118.3
O5—C5—C4	123.46 (11)	C18—C17—H17	118.3
O6—C5—C4	112.56 (10)	C17—C18—C19	119.56 (12)
O4—C6—O3	125.98 (11)	C17—C18—H18	120.2
O4—C6—C3	121.77 (11)	C19—C18—H18	120.2
O3—C6—C3	112.24 (10)	C20—C19—C18	116.91 (11)
C7—N1—C16	117.68 (11)	C20—C19—C19^i^	121.93 (14)
C13—N2—C12	117.39 (12)	C18—C19—C19^i^	121.17 (13)
N1—C7—C8	123.27 (12)	C21—C20—C19	119.66 (12)
N1—C7—H7	118.4	C21—C20—H20	120.2
C8—C7—H7	118.4	C19—C20—H20	120.2
C7—C8—C9	119.50 (12)	N3—C21—C20	123.26 (13)
C7—C8—H8	120.2	N3—C21—H21	118.4
C9—C8—H8	120.2	C20—C21—H21	118.4
C8—C9—C15	117.13 (11)		
			
O2—C1—C2—C3	5.62 (19)	C8—C9—C10—C11	−17.8 (2)
O1—C1—C2—C3	−174.98 (11)	C15—C9—C10—C11	163.88 (13)
C1—C2—C3—O7	66.37 (13)	C14—C10—C11—C12	0.6 (2)
C1—C2—C3—C4	−176.64 (10)	C9—C10—C11—C12	178.46 (13)
C1—C2—C3—C6	−54.58 (14)	C13—N2—C12—C11	−0.1 (2)
O7—C3—C4—C5	−52.92 (13)	C10—C11—C12—N2	−0.3 (2)
C2—C3—C4—C5	−171.64 (10)	C12—N2—C13—C14	0.2 (2)
C6—C3—C4—C5	66.59 (13)	N2—C13—C14—C10	0.1 (2)
C3—C4—C5—O5	−56.85 (17)	C11—C10—C14—C13	−0.5 (2)
C3—C4—C5—O6	123.21 (12)	C9—C10—C14—C13	−178.34 (13)
O7—C3—C6—O4	4.99 (16)	C8—C9—C15—C16	−1.8 (2)
C2—C3—C6—O4	127.10 (12)	C10—C9—C15—C16	176.56 (13)
C4—C3—C6—O4	−114.14 (13)	C7—N1—C16—C15	1.7 (2)
O7—C3—C6—O3	−174.06 (10)	C9—C15—C16—N1	0.0 (2)
C2—C3—C6—O3	−51.95 (14)	C21—N3—C17—C18	−0.6 (2)
C4—C3—C6—O3	66.82 (13)	N3—C17—C18—C19	−0.1 (2)
C16—N1—C7—C8	−1.6 (2)	C17—C18—C19—C20	0.8 (2)
N1—C7—C8—C9	−0.3 (2)	C17—C18—C19—C19^i^	−178.91 (15)
C7—C8—C9—C15	2.0 (2)	C18—C19—C20—C21	−0.8 (2)
C7—C8—C9—C10	−176.40 (13)	C19^i^—C19—C20—C21	178.91 (15)
C8—C9—C10—C14	159.96 (13)	C17—N3—C21—C20	0.7 (2)
C15—C9—C10—C14	−18.3 (2)	C19—C20—C21—N3	0.1 (2)

Symmetry codes: (i) −*x*+2, −*y*, −*z*+2.

### Hydrogen-bond geometry (Å, °)

**Table:**

*D*—H···*A*	*D*—H	H···*A*	*D*···*A*	*D*—H···*A*
O1—H1A···N3^ii^	0.84	1.80	2.6275 (14)	168
O3—H3A···N1^iii^	0.84	1.75	2.5880 (14)	173
O6—H6A···N2^iv^	0.84	1.82	2.6443 (14)	168
O7—H7A···O4	0.84	2.22	2.6538 (13)	112
C2—H2A···Cg^v^	0.99	2.63	3.5579 (13)	160

Symmetry codes: (ii) *x*+1, *y*, *z*; (iii) *x*+1/2, −*y*+1/2, *z*+1/2; (iv) *x*, *y*, *z*−1; (v) *x*−1, *y*, *z*.
